# Relationship between Advanced Glycation End Products and Steroidogenesis in PCOS

**DOI:** 10.1186/s12958-016-0205-6

**Published:** 2016-10-21

**Authors:** Deepika Garg, Zaher Merhi

**Affiliations:** 1 Department of Obstetrics and Gynecology, Maimonides Medical Center, Brooklyn, NY 11219 USA; 2 Division of Reproductive Biology, Department of Obstetrics and Gynecology, NYU School of Medicine, 180 Varick Street, sixth floor, New York City, NY 11014 USA

**Keywords:** Advanced glycation end products (AGEs), RAGE, PCOS, Steroidogenesis, Cholesterol side-chain cleavage enzyme, Steroidogenic acute regulatory protein (StAR), 17α-hydroxylase, 3β-hydroxysteroid dehydrogenase, Aromatase

## Abstract

**Background:**

Women with PCOS have elevated levels of the harmful Advanced Glycation End Products (AGEs), which are highly reactive molecules formed after glycation of lipids and proteins. Additionally, AGEs accumulate in the ovaries of women with PCOS potentially contributing to the well-documented abnormal steroidogenesis and folliculogenesis.

**Main body:**

A systematic review of articles and abstracts available in PubMed was conducted and presented in a systemic manner. This article reports changes in steroidogenic enzyme activity in granulosa and theca cells in PCOS and PCOS-models. It also described the changes in AGEs and their receptors in the ovaries of women with PCOS and presents the underlying mechanism(s) whereby AGEs could be responsible for the PCOS-related changes in granulosa and theca cell function thus adversely impacting steroidogenesis and follicular development. AGEs are associated with hyperandrogenism in PCOS possibly by altering the activity of various enzymes such as cholesterol side-chain cleavage enzyme cytochrome P450, steroidogenic acute regulatory protein, 17α-hydroxylase, and 3β-hydroxysteroid dehydrogenase. AGEs also affect luteinizing hormone receptor and anti-Mullerian hormone receptor expression as well as their signaling pathways in granulosa cells.

**Conclusions:**

A better understanding of how AGEs alter granulosa and theca cell function is likely to contribute meaningfully to a conceptual framework whereby new interventions to prevent and/or treat ovarian dysfunction in PCOS can ultimately be developed.

## Background

Polycystic ovary syndrome (PCOS) is one of the most common endocrine disorders in reproductive-aged women [1] causing hyperandrogenism (clinical and/or biochemical), and oligo/anovulation [2, 3]. Hyperandrogenism could be responsible for some of the clinical features of PCOS such as hirsutism [4]. Although the exact mechanism of hyperandrogenism in PCOS is not clear, it is well known that ovaries of women with PCOS have alterations in steroidogenesis at multiple enzymatic levels [5]. Androgen production in women normally occurs in the ovaries, adrenals, and peripheral tissues such as adipose tissue and skin due to conversion of androgen precursors [6, 7]. The ovaries and the adrenal glands account for half of the androstenedione and T production while adrenal glands account for the majority of DHEAS [8, 9]. In PCOS, elevation in serum androgens such as dehydroepiandrosterone (DHEA), DHEA sulfate (DHEAS), androstenedione, and testosterone (T) is observed due to abnormally increased ovarian and adrenal production [10–15]. Elevation in androgen levels has been reported in 60–80 %, while elevation in serum DHEAS alone has been reported in approximately 20–30 % in women with PCOS [8, 16]. This could be due to the increased production and/or reduced clearance of androgens in PCOS [17].

**Fig. 1:**
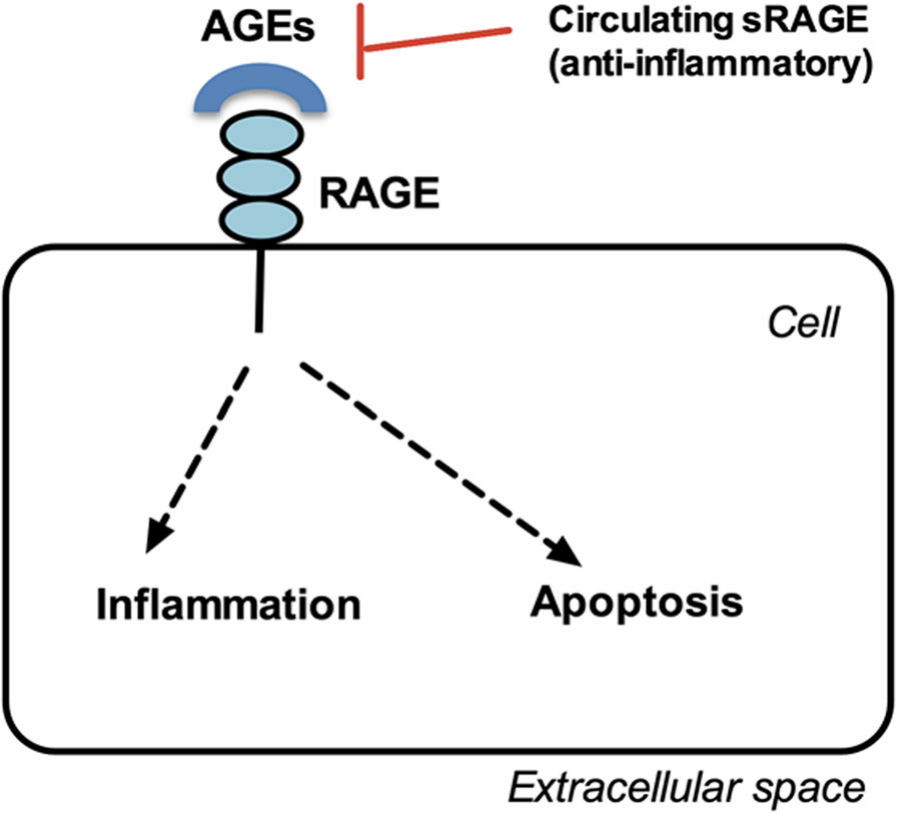
Schematic diagram of the pathogenetic effects of AGEs. AGEs could damage cellular structures via formation of cross-links between key molecules in the basement membrane of the extracellular matrix. The interaction with the cell membrane receptor RAGE induces intracellular inflammation and apoptosis. The circulating receptor for AGEs (sRAGE) acts as decoy by binding the circulating AGEs, thus conferring a potential protective role

One of the molecules that are elevated in women with PCOS is Advanced Glycation End Products (AGEs) [18, 19]. AGEs, also called “glycotoxins,” are the byproducts of Maillard reaction in which the carbonyl group of carbohydrates non-enzymatically interacts with lipids or with the amino group in proteins [20]. AGEs can be formed endogenously by the body or absorbed exogenously by intake of diet containing high levels of AGEs (such as fast-food diet) or by smoking [21]. AGEs are known to play a role in the pathogenesis of different diseases such as diabetes, aging, Alzheimer disease, atherosclerosis, renal disease, and recently PCOS by causing oxidative stress, altering enzymatic activities, affecting cytotoxic pathways, or damaging nucleic acids [19, 22, 23]. AGEs can exert their effect via receptor-independent pathway (for example by binding to the extracellular matrix in many organs) or via receptor-dependent pathway by interacting with their cellular membrane receptor RAGE [24]. Another receptor called soluble receptor for AGEs (sRAGE) circulates throughout the body and is considered a decoy because it binds AGEs and prevents their binding to RAGE thus attenuating their inflammatory effects on bodily tissues [25, 26] (Fig. 1). Data have shown that serum levels of AGEs and the expression of their proinflammatory RAGE are elevated in the ovarian tissue of women with PCOS, potentially altering steroidogenesis and folliculogenesis [27, 28]. The purpose of this review is to summarize the alterations in steroidogenesis that occur in women with PCOS at the level of several enzymes in granulosa and theca cells, and to present data in support of the hypothesis that elevations in systemic AGEs could be partly responsible for PCOS-related alterations in steroidogenesis.

## Material and methods

### Data extraction, inclusion criteria, search strategy, and outcome measures

A systematic review of basic science (in vitro and in vivo) and clinical studies (retrospective and prospective) available in PubMed through June 2016 was conducted. We used the following search terms: “PCOS,” “steroidogenesis,” “enzyme,” “hyperandrogenism,” “hormone,” “AGEs,” “RAGE,” and “soluble receptor for AGEs (sRAGE), cholesterol side-chain cleavage enzyme cytochrome P450 (CYP11A1 or P450scc), steroidogenic acute regulatory protein (StAR), 17α-hydroxylase (CYP17A1), 3β-hydroxysteroid dehydrogenase (3β-HSD), and aromatase.” The references from the related manuscripts were reviewed. All the abstracts of citations from relevant articles were checked. The reviewers considered the abstracts and full text articles from the citations if they were found potentially relevant to steroidogenesis and PCOS. Data extraction was done from the text, graphs, and tables of the identified articles. Additional references were searched from the reference list of the relevant articles. Data abstraction was done and presented in a systemic manner. The main outcomes included changes in steroidogenic enzyme activity at the level of theca and granulosa cells.

## Results

### Alteration in steroidogenesis in PCOS

The changes at the molecular level in granulosa and theca cells of polycystic ovaries are poorly understood. Women with PCOS generally have elevated gonadotropin-releasing hormone (GnRH) pulsatile activity, high levels of luteinizing hormone (LH), hyperactivity of theca-stromal cell and altered activity of granulosa cell that causes reduced production of estradiol (E2) and progesterone (P4) by pre-ovulatory follicle [29, 30]. Sander et al. [31] reported lower levels of E2 and P4 in spite of hyper-luteinized milieu in the ovarian follicles of women with PCOS. In the theca cells, it has been proposed that there is an increased expression of certain alleles that are responsible for the expression of steroidogenic enzymes [32, 33].

Jakimiuket al [34] studied the genetic basis of receptors in granulosa and theca cells of polycystic ovaries and reported higher mRNA expression levels of LH receptor (LHR), StAR, CYP11A1, and CYP17A1. Doldi et al. [35] compared in vivo and in vitro E2 and P4 levels in women with and without PCOS undergoing in vitro fertilization (IVF) and demonstrated higher serum levels of E2 and P4 (*P* < 0.01 and *P* < 0.05; respectively) in women with PCOS on the day of human chorionic gonadotrophin (HCG) injection trigger. While, after HCG stimulation, in vitro P4 level was increased in granulosa cells of the control group in comparison to granulosa cells of the PCOS group; however, E2 levels were similar in both groups. Additionally, Catteau-Jonard et al. [36] showed dysregulation of granulosa cells in addition to intrinsic dysfunction leading to hyperandrogenism and increased expression of FSH receptor (FSHR) and androgen receptor (AR) in stimulated ovaries of women with PCOS. On the other hand, Almahbobi et al. [37] studied the function of granulosa cells from normal and polycystic ovaries and found that E2 production in response to FSH stimulation in polycystic ovaries was not different than normal ovaries suggesting no intrinsic abnormalities of granulosa cells in the polycystic ovaries.

**Fig. 2:**
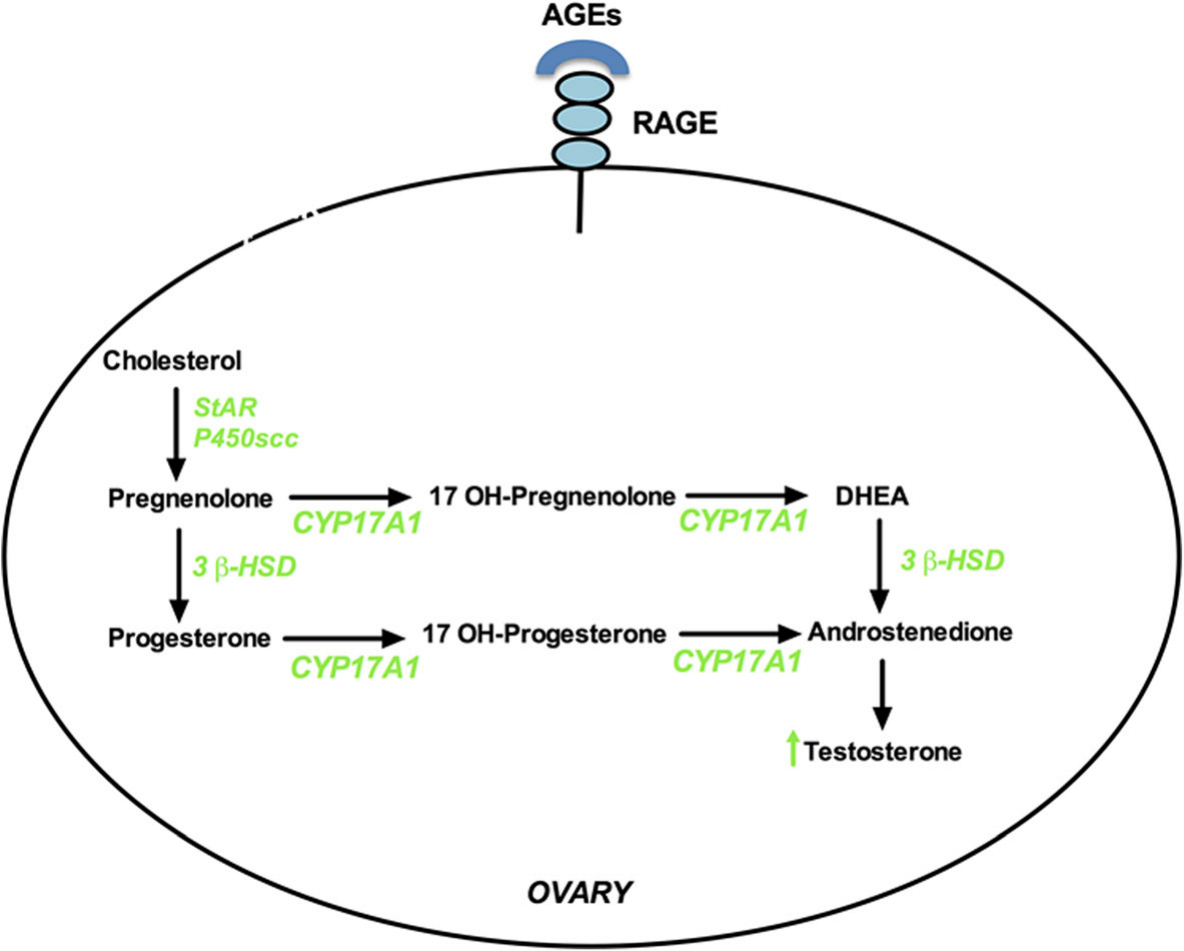
Potential effect of AGE-RAGE binding on steroidogenic enzymes. The binding of AGEs to RAGE induces several steroidogenic enzymes which might lead to increase in testosterone production. Green color represents induction. *StAR: Steroidogenic Acute Regulatory protein, P450scc: cholesterol side chain cleavage enzyme, CYP17A1: 17 alpha-hydroxylase and 17, 20 lyase, and 3β-HSD: 3β hydroxysteroid dehydrogenase*

The pathway of all steroid hormone synthesis begins with the conversion of cholesterol to pregnenolone by CYP11A1 [38, 39] (Fig. 2). Conversion of pregnenolone to 17-hydroxypregnenolone and P4 to 17-hydroxyprogesterone (17 OH-P) is mediated by CYP17A1 which has dual functions of both 17 α-hydroxylase and 17, 20-lyase [40]. Then, 17-hydroxypregnenolone is converted into dehydroepiandrosterone (DHEA) by the 17,20 lyase activity of the same enzyme. DHEA is converted to androstenendione by 3β-HSD enzyme [5]. Various studies have inspected the activity of the enzymes involved in the biosynthesis of steroid hormones in PCOS [41, 42] (Table 1). The production of P4, 17 OH-P, DHEA, androstenedione, and T increase in LH-stimulated theca cells in a dose-dependent manner [43]. The following steroidogenic enzymes will be discussed here in this manuscript:

**Table 1:** Changes in steroidogenesis observed in PCOS and PCOS models

Study	Subjects, animals, or cell line	Intervention/gene quantification	Outcome
Li et al., 2013 [46]	- PCO-like hyper androgenic rat (Sprague Dawley rat) model induced by insulin and hCG injections	- P450scc (CYP11A1) and CYP17A1 gene expression in ovarian theca cells - Ovarian morphology - Changes in estrous cycle - Ovarian androgen production	- Elevated expression of P450scc in thecal and stromal cells - Elevated CYP17A1 gene expression - Reduced granulosa cell layers and augmented theca cell layers with multiple large cysts - Abnormal estrous cyclicity - Elevated androstenedione and T levels
Franks et al., 1997 [32]	- Symptomatic women with PCOS (polycystic ovaries on ultrasound and symptoms) (*n* = 97) - Asymptomatic PCOS (polycystic ovaries on ultrasound and no symptoms) (*n* = 51) - No PCOS with normal ovaries on ultrasound (*n* = 59)	- Genotype analysis of CYP11A1 using microsatellite marker in the promoter region - Non-parametric linkage analysis in CYP11A1 region using polymorphic markers	- Excess allele sharing/linkage at the CYP11A1 locus with a non-parametric linkage score of 3.03 (probability of CYP11A1 gene being linked to PCOS) - CYP11A1 as a major genetic susceptibility locus for PCOS
Wickenheisser et al., 2012 [44]	- Women with PCOS (*n* = 5), defined by NIH consensus guidelines - Women without PCOS (*n* = 5)	- CYP11A1 gene expression at transcriptional and post-transcriptional level using RT-PCR, mRNA degradation studies, and functional promoter analyses - Basal and cAMP-dependent CYP11A1 promoter function - CYP11A1 mRNA half-life - Expression of CYP17A1 gene	- Increased CYP11A1 promoter activity and steady state CYP11A1 mRNA abundance in both basal and forskolin stimulated conditions in PCOS theca cells - More than 2-fold increase in CYP11A1 mRNA half-life - Increased basal mRNA stability in PCOS ovarian theca cells - Increased expression of CYP17A1 gene at both transcriptional and post-transcriptional level in PCOS
Liu et al., 2011 [45]	- Women with PCOS (*n* = 12) who underwent laparoscopic ovarian wedge resection - Control women (*n* = 12) who underwent contralateral ovarian biopsy	- Expression of CYP11A1 mRNA and protein levels using RT-PCR and Western blot analyses	- Higher CYP11A1mRNA and protein levels in PCOS group
Sander et al., 2011 [31]	- Women with (*n* = 28) PCOS and without (*n* = 28) PCOS who underwent IVF	- CYP11A1 mRNA expression levels in luteinized granulosa cells - Follicular fluid E2 and P4 levels - StAR and 3β-HSD mRNA expression in granulosa cells	- No change in CYP11A1 mRNA expression levels in granulosa cells between two groups - Significantly lower E2 and P4 levels in the follicular fluid of women with PCOS - Increased expression of StAR mRNA levels in women with PCOS - No change in 3β-HSD mRNA expression in polycystic ovaries
Hogg et al., 2012 [49]	- 11 months-old female offspring of ewe PCO-model induced by prenatal testosterone propionate (TP) (*n* = 9) - Age-matched control ewe (*n* = 5)	- StAR gene expression - Androstenedione levels	- Enhanced androstenedione secretion in the antral follicles of PCO-like ewe that was augmented after treatment with recombinant LH - Higher thecal LH receptor gene expression - Increased number of estrogenic follicles - Up-regulation in StAR mRNA expression in theca cells
Jakimiuk et al., 2001 [34]	- Women with PCOS (*n* = 12) undergoing electrocauterization of the ovarian surface or wedge resection of the ovaries, - Age-matched control (*n* = 24) premenopausal women undergoing total abdominal hysterectomy with bilateral oophorectomy for non-ovarian indications	- Expression of StAR, CYP17A1, CYP11A1 and LH receptor mRNA	- Elevated expression of StAR, CYP17A1, CYP11A1, and LH receptor mRNA in the theca cells of women with PCOS - Increased LH receptor and CYP11A1 expression in granulosa cells of women with PCOS
Kahsar-Miller et al., 2001 [50]	- Women with PCOS (*n* = 7) - Control women (*n* = 10)	- StAR expression - Ovarian morphology	- Significantly higher number of follicular cysts and staining for StAR immunoreactivity in theca cell of women with PCOS
Nelson et al., 1999 [51]	- Normal and PCOS theca interna cells	- CYP17A1, CYP11A1, and 3β-HSD mRNA and protein levels - P4, 17OHP, and T levels	- Increased production of P4, 17OHP, and T by theca cells of women with PCOS - Elevated CYP17A1, CYP11A1, and 3β-HSD mRNA and protein levels in theca cells of women with PCOS - No difference in StAR mRNA expression
Wickenheisser et al., 2000 [55]	- Theca cells of women with or without PCOS	- CYP17A1 and StAR promoter activity	*In PCOS theca cells:* - Four-fold greater CYP17A1 promoter activity - Augmented basal and cAMP-dependent CYP17A1 gene expression - Slower degradation of CYP17A1 mRNA - No change in StAR promoter activity
Doldi et al., 2000 [57]	- Women with (*n* = 10) and without (*n* = 10) PCOS	- 3β-HSD mRNA expression	- Lower expression of 3β-HSD mRNA in PCOS granulosa cells obtained from follicles measuring ≤ 10 mm and > 16 mm
Erickson et al., 1979 [70]	- Granulosa cells from normal and polycystic ovaries	- Ability of granulosa cells to aromatize androgens after in vitro incubation of granulosa cells with androstenedione, FSH and LH	- Elevated E2 production in granulosa cells from normal (8–15 mm) follicles - Negligible E2 production by granulosa cells from small (4–6 mm) follicles of both normal and polycystic ovaries - 17–24 fold increase in E2 production in PCOS and control ovaries in response to FSH - Little or no effect on E2 production by addition of LH
Pierro et al., 1997 [64]	- Granulosa luteal cells from polycystic and normal ovaries	- Effect of atamestane (aromatase inhibitor) on granulosa luteal cells	- Robust inhibition of basal aromatase activity after treatment with atamestane in both groups with more pronounced effect in cells of normal ovaries in comparison to cells of PCOS ovaries
Andreani et al., 1994 [71]	- Granulosa cells from polycystic ovaries and normal ovaries in the preovulatory phase after oocyte retrieval during GIFT	- E2 and P4 production in the presence or absence of FSH	- 2–3 fold increase in E2 production in PCOS ovaries after FSH treatment - No change in P4 production after FSH treatment
Mason et al., 1994 [65]	- Granulosa cells from normal, ovulatory and anovulatory polycystic ovaries	- FSH-induced E2 production - Follicular androstenedione levels	- Significantly higher androstenedione in small follicles (5–11 mm) from ovulatory PCOS - 6–10 times higher FSH-induced E2 response in anovulatory PCOS
Söderlund et al., 2005 [72]	- Women with PCOS (*n* = 25), - Women without PCOS (*n* = 50)	- PCR analysis of genomic DNA and complete sequence of all exons of the aromatase gene and its promoter	- Mutations of the P450arom gene or its promoter were not found to be associated with PCOS

*Abbreviations*: *PCOS* polycystic ovary syndrome, *FSH* Follicle-stimulating hormone, *LH* luteinizing hormone, P450scc cholesterol side-chain cleavage enzyme, *StAR* steroidogenic acute regulatory protein, *3β-HSD* 3beta-hydroxysteroid dehydrogenase, *CYP17A1* 17α-hydroxylase, *hCG* human chorionic gonadotropin, *T* testosterone, *IVF* in vitro fertilization, *GIFT* gamete intra-fallopian transfer, *E2* estradiol, *P4* progesterone, *RT-PCR* reverse transcription-polymerase chain reaction, *17OHP* 17-hydroxyprogesterone

#### P450scc (CYP11A1)

CYP11A1 regulates the first step of steroidogenesis and forms pregnenolone from cholesterol [39]. In polycystic ovaries, there seems to be an alteration in the CYP11A1 gene expression. For instance, Franks et al. [32] described the role of CYP11A1 encoding gene in the pathogenesis of excess androgen production in women with polycystic ovaries. Their data from both association and linkage studies suggested that CYP11A1 is a major genetic susceptibility locus for PCOS. They examined the segregation of CYP11A1 in 20 families and performed association studies in premenopausal women with polycystic ovaries and matched control women from a similar ethnic background. Using a microsatellite marker in the promoter region of CYP11A1, they performed genotype analysis after PCR amplification. Their results demonstrated that differences in expression of CYP11A1 could account for variation in androgen production in women who have polycystic ovaries. Using polymorphic markers in the region of CYP11A1, they carried out non-parametric linkage analysis and found evidence for excess allele sharing at the CYP11A1 locus.

Ovarian theca cells isolated from PCOS follicles and maintained in culture produce elevated levels of P4 and androgen compared to theca cells of women without PCOS [44]. Wickenheisser et al. [44] evaluated CYP11A1 gene at transcriptional and post-transcriptional level by quantitative RT-PCR, promoter functional analyses, and degradation studies of mRNA in theca cells of normal and polycystic human ovaries placed in long-term culture. The investigators demonstrated that basal and forskolin-stimulated steady state CYP11A1 mRNA abundance and CYP11A1 promoter activities were significantly increased in PCOS theca cells (Table 1). They also showed that CYP11A1 mRNA half-life increased more than two-folds in PCOS theca cells. These data suggest that elevated CYP11A1 mRNA abundance in PCOS cells results from increased transactivation of the CYP11A1 promoter and increased CYP11A1 mRNA stability. Similarly using RT-PCR, Western blot, and immunohistochemistry, Liu et al. [45] examined the expression of CYP11A1 in follicles in their early and late stages of development in women with and without PCOS who underwent laparoscopic ovarian wedge resection. They reported higher CYP11A1 mRNA and protein levels in early-stage follicles of women with PCOS. These changes could be in part responsible for the changes observed in follicular development in polycystic ovaries. In Sprague Dawley rat model, Li et al. [46] used a hyperandrogenic PCO-like induced by insulin and HCG injections to investigate changes in ovarian CYP11A1 expression (Table 1). Using Western blot and immunohistochemistry, they reported increased expression of CYP11A1 in theca cells as well as abnormal estrous cyclicity, increased ovarian weight/body weight ratio, elevated ovarian androgen production (androstenedione and T) with reduced number of granulosa cell layers and increased number of theca cell layers compared to the control rats [46]. One of the drawbacks of that study is that insulin and HCG trigger a PCO-like phenotype that is not similar to PCOS phenotype in humans.

One the other hand, not all studies has shown upregulation in CYP11A1 (Table 1). For instance, Sander et al. [31] compared CYP11A1 mRNA expression levels in granulosa cells extracted from women with or without PCOS who underwent controlled ovarian hyperstimulation for IVF and reported no changes between both groups of women. They reported that women with PCOS had significantly lower E2 and P4 levels in their follicular fluid. One drawback of that study is that it evaluated mRNA levels in luteinized granulosa cells that were exposed to high doses of gonadotropins. Additionally, CYP11A1 mRNA levels were not measured in theca cells.

#### StAR

StAR protein helps in the transport of cholesterol from the cell membrane into the mitochondria leading to the initiation of the steroidogenic pathway [47, 48]. Sander et al. [31] reported an increased expression of StAR mRNA levels in granulosa cells collected from women with PCOS who underwent controlled ovarian hyperstimulation for IVF compared to women without PCOS. Hogg et al. [49] conducted a study on ewe PCO-model induced by prenatal T propionate (TP) administration to pregnant ewe. The offspring females were sacrificed at 11 months of age after synchronization of the estrous cycles by administration of prostaglandin to ensure that they were in the follicular phase. Their ovaries were assessed for steroidogenic gene expression. Both in vivo and in vitro, enhanced androstenedione secretion was observed in the antral follicles of TP-treated PCO-like ewe, more so after treatment with recombinant LH. Augmented thecal LHR gene expression was also present in these animals. Additionally, there was up-regulation in StAR (*P* < 0.01), CYP11A1 (*P* < 0.05), CYP17A1 (*P* < 0.05), and 3β-HSD (*P* < 0.01) mRNA expression levels in theca cells. This indicates that the ovarian programming induced by prenatal androgens in animals could cause hypersecretion of androgens by altering LH sensitivity and up-regulation of steroidogenic genes in theca cells.

Jakimiuk et al. [34] investigated the gene expression of steroidogenic enzymes including StAR from granulosa and theca cells of women with PCOS (*n* = 12) under age 44 years undergoing electrocauterization of the ovarian surface or wedge resection of their ovaries for infertility treatment and control (*n* = 24). Participants in both groups were age-matched premenopausal women undergoing total abdominal hysterectomy with bilateral oophorectomy for non-ovarian indications. Granulosa cells were obtained from both the follicular fluid (after centrifugation) and the wall of the follicles (by scraping). Theca cells were collected from the follicle after microdissection. That study showed that in theca cells, expression of StAR, CYP17A1, CYP11A1 and LHR mRNA were significantly higher in PCOS follicles in comparison to the size-matched control follicles. This indicates that theca cells in PCOS are hyperstimulated thus they produce excessive amounts of androgens. That study also showed that in granulosa cells, LHR and CYP11A, but not StAR, mRNA expression was higher in PCOS than in control follicles indicating that granulosa cells in PCOS have increased LH responsiveness that may contribute to arrested follicle development.

On the other hand, some studies showed no changes in StAR expression in PCOS women. For instance, the distribution of StAR in theca cells of women with PCOS (*n* = 7) and healthy females (*n* = 10) was studied by Kahsar-Miller et al. [50]. They assessed StAR expression by immunohistochemistry and Western blot in polycystic and normal ovaries. Although they reported significantly greater number of follicular cysts with theca cell staining for StAR immunoreactivity in women with PCOS, the distribution of StAR immunoreactivity within most of the ovarian structures (including granulosa and theca cells) was not different in the ovaries of women with PCOS compared to ovaries of healthy women. Another study conducted by Nelson et al. [51] evaluated the steroidogenesis in human theca interna cells stripped from ovarian follicle walls of women who underwent hysterectomy. They demonstrated that increased production of P4, 17-OHP, and T by PCOS theca cells propagated in long-term culture (with or without forskolin) was due to increased mRNA and protein activity of CYP17A1, CYP11A1, and 3β-HSD but not StAR enzyme (Table 1). This indicates that the intrinsic defect in the steroid biosynthesis in theca cells at genetic level is not due to overall differences in the regulation of cAMP or adenylate cyclase (activated by forskolin) but due to selective alterations in steroidogenic enzyme expression.

#### CYP17A1

CYP17A1 is an enzyme that belongs to cytochrome P450 superfamily. It catalyzes the activities of both 17 α-hydroxylase and 17, 20 lyase [52]. CYP17A1 is elevated in the ovaries of women with PCOS, which is partly responsible for the altered steroidogenesis in these women [51, 53]. As mentioned earlier (under StAR section), Nelson et al. [51] demonstrated that CYP17A1 enzyme expression and activity is elevated in the human theca interna in culture. As mentioned earlier (under CYP11A1 section), Li et al. [46] demonstrated in a hyperandrogenic PCO-like rat model induced by insulin and HCG injections that the expression of CYP17A1 gene was significantly higher in theca cells compared to control rats.

Wickenheisser et al. [54] (details on the study are described above, under CYP11A1 section) demonstrated increased expression of CYP17A1 gene at both transcriptional and post-transcriptional level in theca interna cells of human polycystic ovaries. In another experiment, Wickenheisser et al. [55] in transfected theca cells with a series of CYP17A1 reporter constructs and showed that cotransfection of steroidogenic factor-1 (SF-1) was required for the detection of both basal and stimulated promoter CYP17A1 activity. Additionally, there was a 4-folds greater CYP17A1 promoter activity in PCOS theca cells compared to normal theca cells. PCOS theca cells had augmented basal and cAMP-dependent CYP17A1 gene expression and there was slower degradation of CYP17A1 mRNA (Table 1). These data suggest that dysregulation of the processes involved in CYP17A1 transcription in PCOS theca cells may, in part, account for the increased ovarian androgen production.

#### 3β-HSD

3β-HSD is an enzyme that catalyzes the conversion of pregnenolone to P4, the conversion of 17-hydroxypregnenolone to 17-OHP, and the conversion of DHEA to androstenedione. It belongs to the family of oxidoreductase and exists as two isozymes: 3β-HSD I and II [56]. Isozyme II exists in the ovary and the adrenal glands and it contributes to androgen biosynthesis. Evidence showed conflicting results related to 3β-HSDII activity in polycystic ovaries with increase, decrease, or no change in its mRNA expression and enzymatic activity [31, 51, 57].

Nelson et al. [51] (details are provided under StAR section above) showed elevated 3β-HSD activity per theca cell under both basal and forskolin-stimulated conditions. Doldi et al. [57] evaluated 3β-HSD mRNA expression using RT-PCR in luteinizing granulosa cells from follicles of women with PCOS. They reported lower expression of this enzyme in the PCOS granulosa cells obtained from follicles measuring ≤ 10 mm and > 16 mm in comparison to control normal granulosa cells. Their data demonstrated that downregulation of 3β-HSD gene could be responsible for the reduction in P4 synthesis by PCOS granulosa cells. In contrast, Sander et al. [31] reported no change in 3β-HSD mRNA expression in polycystic ovaries. Accumulating evidence suggest the basis of elevated androgens in polycystic ovaries could be due to influence at genetic level involving genes of multiple enzymes and subsequently modification in the signal transduction pathways that regulate steroidogenesis [58].

#### CYP19A1

Aromatase, encoded by CYP19A1 gene, is a microsomal enzymatic complex that catalyzes the formation of estrogens (estrone and E2) from androgens (androstenedione and T) during steroidogenesis [59]. LH stimulates steroidogenesis by producing androgens from theca interna cells, which are converted to E2 in granulosa cells by aromatase enzyme under FSH stimulation [60]. Downregulation of this enzyme has been reported to be partially responsible for the altered steroidogenesis in PCOS [61–63] although some data reported elevated aromatase levels in polycystic ovaries [64, 65].

Understanding granulosa cell aromatase and E2 production in the non-PCOS state is crucial in elucidating changes in aromatase activity and E2 production in PCOS. Foldesi et al. [66] demonstrated in vitro the ability of granulosa-lutein cells to have aromatase activity and the ability of these cells to produce E2 spontaneously without the support of androgens and gonadotropins. The addition of recombinant human FSH (rFSH) to granulosa-lutein cells in culture resulted in increased production of E2 in a dose- and time-dependent manner even in the absence of exogenous androgens. Addition of T caused substantial increase in the basal production of E2 that can be moderated by certain follicular fluid compounds [67, 68]. Origin of the follicular fluid and the size of the follicles might decide the stimulatory or inhibitory action on ovarian E2 production by aromatase [69]. Guet et al. [61] investigated in vitro the role of human dominant follicular fluid in altering aromatase activity. They collected follicular fluid by laparoscopy from the dominant follicle of the ovaries of women with tubal or unexplained infertility before their LH surge. Granulosa cells were collected from the women undergoing IVF. They measured aromatase activity after adding various concentration of follicular fluid and control serum (2.5, 5, 10 and 20 %) to granulosa cells and reported reduced aromatase activity with addition of higher concentration of follicular fluid [61]. This indicates that follicular fluid of dominant follicles contains factors that inhibit aromatase activity of these cells.

Erickson et al. [70] studied both in vivo and in vitro granulosa cell ability of synthesizing E2 in normal and polycystic ovaries. They incubated granulosa cells from both normal and polycystic ovaries with androstenedione and measured E2 concentration. They found negligible amounts of E2 production by granulosa cells of small follicles (4–6 mm) from both PCOS and control ovaries. However after culture of both PCOS and control granulosa cells with FSH (100 ng/mL) or LH (100 ng/mL) for 48 h, 24 and 17 times increase in E2 production was noted in response to FSH in both groups respectively with slight or no effect of LH. They also reported elevated E2 production in granulosa cells obtained from granulosa cells of follicles measuring 8–15 mm in the control group. They suggested that granulosa cells from polycystic ovaries have a natural aromatase activity in response to FSH but their follicles never develop to the size (8–15 mm) at which they start producing E2, causing a decline in E2 production in PCOS.

In contrast, Pierro et al. [64] described evidence of an exaggerated in vitro activity of aromatase in granulosa cells of PCOS. They studied the effect of a competitive aromatase inhibitor, atamestane, on granulosa luteal cells obtained from women with or without PCOS following controlled ovarian hyperstimulation. They reported marked inhibition of aromatase activity in granulosa cells from normal ovary in comparison to granulosa cells from polycystic ovaries with lower minimal effective dose of the inhibitor and higher inhibitory effect in normal ovaries suggesting that there was a higher basal aromatase activity in PCOS. Another study demonstrated that aromatase is more sensitive and responsive to FSH in granulosa luteal cells of polycystic ovaries in comparison to normal ovaries [71]. Similar findings were confirmed by Mason et al. [65] who described an enhanced aromatase activity in granulosa cells of polycystic ovaries. They evaluated FSH-induced E2 production in granulosa cells in normal and ovulatory or anovulatory polycystic ovaries. There were no significant differences in FSH or E2 in follicular fluid, but significantly higher androstenedione in small follicles (5 to 11 mm) from ovulatory PCOS compared to normal women. Also, there was 6–10 times more FSH-induced E2 response in anovulatory PCOS compared to controls. Söderlund et al., [72] investigated genomic DNA after PCR analysis from follicles of polycystic ovaries and found that CYP19A1 gene or its promotor mutation was not associated with reduced activity of aromatase in polycystic ovaries (Table 1).

### Role of AGEs in altering steroidogenesis in PCOS

**Fig. 3:**
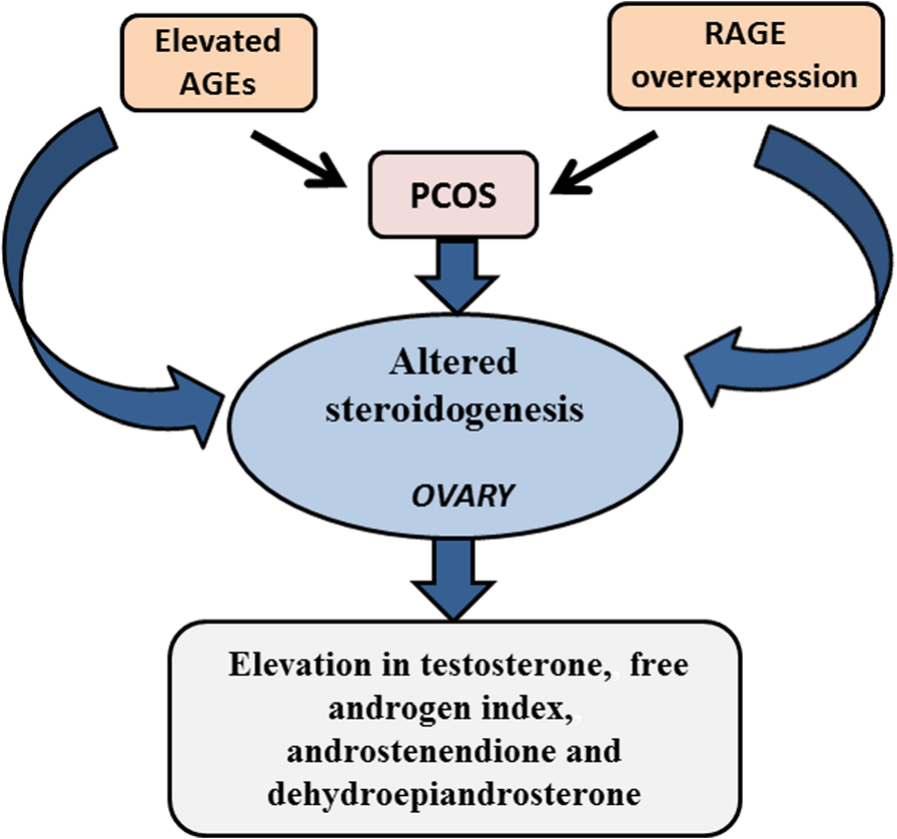
Relationship between AGEs and hyperandrogenism in PCOS. The elevation in serum AGEs and the overexpression of ovarian RAGE in PCOS are associated with hyperandrogenism. *AGEs, advanced glycation end products; RAGE, receptor for advanced glycation end products*

A possible role of AGEs in altering steroidogenesis in PCOS, directly or indirectly, is still unclear and under investigation (Fig. 3). A recent study showed that AGEs such as toxic AGE (TAGE), pentosidine and carboxymethyl lysine (CML) in follicular fluid and serum TAGE (S-TAGE) correlated negatively with E2 levels (follicular fluid pentosidine, *r* = -0.29, *p* < 0.001; follicular fluid CML, *r* = -0.31, *p* < 0.0001; follicular fluid TAGE, *r* = -0.26, *p* < 0.01; and S-TAGE, *r* = -0.25, *p* < 0.01) in PCOS patients undergoing assisted reproductive technology [73]. On immunohistochemical localization of normal and PCOS ovaries, staining for AGEs was highest in endothelial cells and granulosa cell layer of PCOS ovaries [74].

The abnormal steroidogenesis in PCOS could lead to elevated androgen synthesis and abnormal follicular development [75]. AGEs are associated with hyperandrogenemia in PCOS (Fig. 1, Fig. 2, and Table 2). Diamanti- Kandarakis et al. [27] investigated serum AGEs levels and RAGE expression in monocytes of PCOS (*n* = 29) and control (*n* = 22) group and their correlation with T levels. They demonstrated higher serum AGEs protein levels (U/mL) (mean ± SE: 9.81 ± 0.16 vs. 5.11 ± 0.16, *P* < 0.0001), and elevated expression of RAGE (% + ve) (mean ± SE: 30.91 ± 10.11 vs. 7.97 ± 2.61, *P* < 0.02) in patients with PCOS compared to control group. In addition, a positive correlation was observed between serum AGEs and T (*r* = 0.73, *P* < 0.0001), even after controlling for BMI (partial correlation coefficient = 0.61, *P* = 0.0001). In another study, the same authors fed Wistar rats diet containing high (H-AGE) or low (L-AGE) AGEs for 6 months. In H-AGE rats, they found elevated AGE deposition in ovarian theca interna cells (*P* = 0.003), increased RAGE staining in granulosa cells (*P* = 0.038), and higher plasma T (*P* < 0.001) compared to L-AGE rats [76]. In addition, Tantalaki et al. [77] investigated the role of AGEs dietary intake on hormonal status of women with PCOS. They gave women with PCOS (*n* = 23) isocaloric diet containing H-AGE or L-AGE for 2 months and found higher serum AGEs (IU/mL) (mean value ± SD: 10.4 ± 1.4 vs 8.2 ± 1.6, *P* < 0.05) along with elevated T, free androgen index (FAI) and androstendione levels (T: 1.04 ± 0.43 (ng/ml) vs 0.77 ± 0.32 (ng/ml), FAI: 15.4 ± 16 vs 11.1 ± 10.7, androstendione: 3.76 ± 1.10 (ng/ml) vs 3.37 ± 0.96 (ng/ml), *P* < 0.05) in PCOS patients with H-AGE diet compared to L-AGE diet. Chatzigeorgiou A et al. [78] fed female Wistar rats H-AGE or L-AGE diet for 3 months and demonstrated elevated levels of plasma T in rats in the H-AGE diet group. Additionally, lower levels of E2 and P4, higher levels of T along with decreased expression of scavenger receptors of AGEs were found in H-AGE diet fed rats compared to L-AGE diet fed animals [78]. These studies substantiate the association between AGEs and hyperandrogenism in PCOS. Accumulating evidence has demonstrated that dietary modification with intake of diet containing low AGEs can reduce the concentration of plasma bioavailable testosterone and can modify PCOS phenotypes [21, 79].

**Table 2:** Studies evaluating the association between AGEs and PCOS

Study	Subjects, animals, or cell line	Intervention	Outcome
Diamanti- Kandarakis et al., 2007 [76]	- Female Wistar rats fed high (H-AGE) or low (L-AGE) diet for 6 months	- AGEs’ levels in ovarian theca cells - RAGE expression - Plasma T level	*In H-AGE diet rats:* - Elevated AGEs’ deposition in ovarian theca interna cells - Increased RAGE staining in granulosa cells - Higher plasma T levels
Chatzigeorgiou A et al., 2013 [78]	- Female Wistar rats fed high (H-AGE) or low (L-AGE) diet for 3 months	- Plasma T, E2 and P4 levels - Expression of scavenger receptors for AGEs	*In H-AGE diet rats:* - High T plasma levels - Lower levels of E2 and P4 - Reduced expression of scavenger receptors for AGEs
Jinno et al., 2011 [73]	- Women with (*n* = 71) and without (*n* = 86) PCOS undergoing ART	- Measurement of toxic AGEs (TAGE), pentosidine, and CML in blood and follicular fluid	- Negative correlation between E2 and follicular fluid AGEs (TAGE, Pentosidine and CML) - Negative correlation between E2 and serum AGEs (TAGE)
Diamanti- Kandarakis et al., 2005 [27]	- Women with (*n* = 29) or without (*n* = 22) PCOS	- Serum AGEs’ levels and RAGE expression in circulating monocytes - Correlation between AGEs and T levels	- Higher serum AGEs’ levels and elevated expression of RAGE in PCOS - Positive correlation between serum AGEs and T
Tantalaki et al., 2014 [77]	- Women with PCOS (*n* = 23) were given isocaloric diet containing high (H-AGE) or low (L-AGE) levels of AGEs for 2 months	- Serum AGEs and androgens (T, and androstendione) levels, free androgen index	*Women with PCOS on H-AGE diet:* - Higher serum AGEs - Elevated T, androstenedione, and free androgen index
Diamanti-Kandarakis et al., 2013 [80]	- Human ovarian granulosa cell line (KGN) treated with recombinant LH in the presence or absence of human glycated albumin (HGA) (representative of AGEs)	- Effect of AGE-RAGE on LH signaling	- Interference of LH actions by ovarian AGEs due to sustained activation of ERK1/2 and MAPK signaling - May also impair follicular responses to hormones
Merhi et al., 2015 [83]	- Cumulus granulosa cells (CCs) (*n* = 6) treated with HGA (representative of AGEs) obtained from women who underwent IVF - KGN granulosa cell line treated with recombinant AMH (rAMH) in the presence or absence of HGA - Follicular fluid levels of sRAGE and AGEs in women undergoing IVF	- mRNA expression of LH receptor (LHR), AMH, AMHR-II, and RAGE by RT-PCR - RAGE protein expression by immunofluorescence - Immunofluorescence for SMAD 1/5/8 phosphorylation (AMH signaling pathway) - Correlation between sRAGE and AGEs (pentosidine and CML) in follicular fluid	- HGA increased LHR and AMHR-II mRNA levels - HGA did not change AMH mRNA levels - HGA increased RAGE protein levels - HGA significantly increased rAMH-induced SMAD 1/5/8 phosphorylation - sRAGE positively correlated with pentosidine and CML

*Abbreviations*: *AGEs* advanced glycation end Products, *RAGE* receptors for advanced glycation end products, *ART* Assisted Reproductive Technology, *CML* carboxymethyl lysine, *E2* Estradiol, *T* testosterone, *AMH* anti-Mullerian hormone, *AMHR-II* AMH receptor, *sRAGE* soluble receptor for AGEs, *IVF* in vitro fertilization

Obesity and insulin resistance are commonly observed in women with PCOS [1]. Recent studies have demonstrated a role for AGEs in the development of obesity [80, 81]. For example in one study, after feeding C57/BL6 and db/db mice with H-AGE or L-AGE diet for 6 months, a significant weight gain was observed in H-AGE diet group [80]. In another study, when C57BL6 mice were fed diet with or without AGEs, increased adiposity was noted in animals fed diet-containing AGEs [81]. Insulin resistant women with PCOS without hyperglycemia have elevated serum levels of AGEs and upregulation in RAGE expression in their circulating monocytes [27]. Additionally, serum AGE levels are positively correlated with testosterone level, free androgen index, insulin, HOMA and waist-to-hip ratio in women with PCOS who did not have hyperglycemia [82]. Another study has shown that increased serum AGE levels is observed in non-insulin resistant lean women with PCOS suggesting that this association is independent on insulin resistance state [28].

**Fig. 4:**
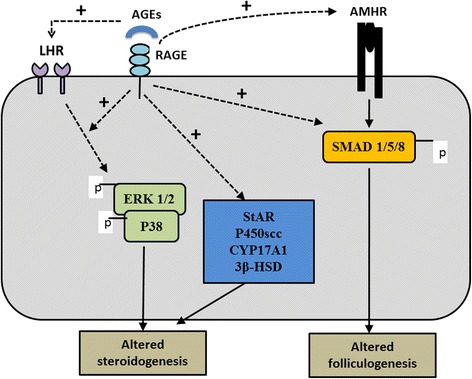
A diagram of pathways by which AGEs affect genes involved in steroid synthesis and follicular development. AGEs induce LHR mRNA expression and alter LH hormone action by inappropriate activation of ERK1/2 pathway. The interaction of AGEs with its cell membrane receptor RAGE induces StAR (Steroidogenic Acute Regulatory protein), P450scc (cholesterol side chain cleavage enzyme), CYP17A1 (17 alpha-hydroxylase and 17, 20 lyase), and 3β-HSD (3β hydroxysteroid dehydrogenase) mRNA expression levels. AGE-RAGE interaction also induces AMHR-II mRNA levels and increases SMAD 1/5/8 phosphorylation (AMH pathway). *AGEs: advanced glycation end-products; RAGE: receptor for advanced glycation end-products; AMH: anti-Mullerian hormone*

The AGE-RAGE interaction leads to downstream signaling cascades that play important role in the RAGE mediated actions of AGEs [83]. Mechanism of AGE-RAGE signaling in altering steroidogenesis in PCOS has been studied by Diamanti-Kandarakis et al. [84] in human ovarian granulosa cells (KGN) in which they cultured KGN granulosa cell line with LH alone and LH along with human glycated albumin (HGA, representative of AGEs) for 0–2 h. They hypothesized that the interference of ERK1/2 activation by AGE-RAGE signaling in human granulosa cells causes attenuation of LH action. The basis of this hypothesis was the involvement and activation of ERK1/2 by both AGE-RAGE intracellular signaling and LH-induced oocyte maturation and ovulation. In granulosa cells LH-induced oocyte maturation and ovulation requires activation of MAPK specifically ERK1/2 pathway [85, 86]. The authors showed that AGEs cause inappropriate activation of ERK1/2 pathway in KGN granulosa cells that could lead to altered steroidogenesis these cells (Fig. 4).

RAGE is a multi-ligand receptor that belongs to the immunoglobulin superfamily. RAGE ligands include AGEs, high mobility group box 1 (HMGB1), S100/calgranulins, Mac-1, phosphatidylserine and amyloid β (Aβ) [87–89]. RAGE acts as an extracellular S100 receptor due to sharing of same structural features with S100/calgranulin [90]. Another proinflammatory multifunctional protein called HMGB1 binds RAGE and acts as a transcriptional regulator for inflammatory response and tumor progression [91–93]. The function of HMGB1 is dependent on its location (i.e., intracellular or extracllular). Intracellular HMGB1 acts as a regulator of transcription by binding to DNA. HMGB1 can be either actively secreted by the cell or passively released by the cell upon its death [91]. Extracellular HMGB1 activates RAGE, which in turn can promote an immune response [94, 95]. Recently, p38-regulated/activated protein kinase (PRAK) has been identified as a novel RAGE ligand [87]. Further studies are required to investigate the better understanding of the involvement of RAGE and its interaction to different ligands in the pathogenesis of PCOS.

Merhi et al. [96] recently studied the adverse effects of AGEs on steroidogenesis and follicular development. The authors measured sRAGE, AGEs (pentosidine and CML) by ELISA in follicular fluid of women who underwent IVF and found positive correlation of sRAGE with pentosidine (*r* = 0.24), and CML (*r* = 0.32) (*p* < 0.05). They measured mRNA for LHR, AMH and its receptor (AMHR-II), and RAGE after culture of cumulus granulosa cells of women who underwent IVF with media ± human glycated albumin (representative of AGEs) for 48 h. They found 59 % increase in LHR, and 52 % increase in AMHR-II mRNA levels (*p* < 0.05) with no change in AMH mRNA levels in HGA treated granulosa cells compared to control (cells treated with media alone). Finally, the authors treated KGN granulosa cell line with recombinant AMH (rAMH; 50 ng/mL) with or without HGA (0.4 ng/mL) and assessed SMAD 1/5/8 phosphorylation (AMH signaling pathway). Recombinant AMH markedly increased the phosphorylation of SMAD 1/5/8 in KGN cells compared to control cells. When KGN cells were pretreated with HGA in the presence of rAMH, the accumulation of phospho-SMAD 1/5/8 in the nucleus was significantly augmented compared to control cells (media alone) and compared to cells treated with rAMH alone. These findings are in alignment to the above changes described in various enzymes involved in steroidogenesis in PCOS and further confirm the role of AGEs in alteration of genes involved in steroidogenesis in PCOS [96].

## Conclusion

PCOS is a state of elevated androgen production, which is caused by alteration in the steroid biosynthesis by affecting different steroidogenic enzymes. Hyperandrogenism is one of the causes of subfertility associated with PCOS. Accumulating evidence has suggested the possibility of underlying role of AGEs in altering steroid bio-synthesis in polycystic ovaries by affecting enzyme function/activity. AGE-induced augmentation of insulin resistance and induction of inflammatory changes in polycystic ovaries are other possible mechanisms that contribute to altered steroidogenesis in polycystic ovaries. If exact mechanism of AGEs induced hyperandrogenism in PCOS is established, the targeted therapy against AGEs will be beneficial to improve the reproductive outcome in this patient population. In summary, AGEs seem to affect ovarian steroid production. Understanding the role of the AGE-RAGE/sRAGE axis in ovarian dysfunction, particularly in women with PCOS who have elevated levels of these AGEs, might shed light for better understanding the mechanisms behind female reproduction.

## Abbreviations

17 OH-P: 17- hydroxyprogesterone
3β-HSD: 3β-hydroxysteroid dehydrogenase
AGEs: Advanced Glycation End Products
AMH: Anti-Mullerian hormone
AR: Androgen receptor
ART: Assisted reproductive technology
CML: Carboxymethyl lysine
CYP17A1: 17α-hydroxylase
DHEA: Dehydroepiandrosterone
E2: Estradiol
FSH: Follicle-Stimulating Hormone
FSHR: FSH receptor
GnRH: Gonadotropin-releasing hormone
HCG: Human chorionic gonadotrophin
IVF: In vitro fertilization
LH: Luteinizing hormone
LH: Luteinizing hormone
P4: Progesterone
P450scc: Cholesterol side-chain cleavage cytochrome P450
PCOS: Polycystic ovary syndrome
RAGE: Receptors for AGEs
rAMH: Recombinant AMH
rFSH: Recombinant human FSH
sRAGE: Soluble receptor for AGEs
StAR: Steroidogenesis acute regulatory protein
T: Testosterone
